# Voluntary and Involuntary Movements Widen the Window of Subjective Simultaneity

**DOI:** 10.1177/2041669517719297

**Published:** 2017-07-07

**Authors:** B. Ezgi Arikan, Bianca M. van Kemenade, Benjamin Straube, Laurence R. Harris, Tilo Kircher

**Affiliations:** Department of Psychiatry and Psychotherapy, Philipps-University Marburg, Germany; Department of Psychology and Centre for Vision Research, York University, Canada; Department of Psychiatry and Psychotherapy, Philipps-University Marburg, Germany

**Keywords:** voluntary action, multisensory, simultaneity perception, time perception

## Abstract

Forming a coherent percept of an event requires different sensory inputs originating from the event to be bound. Perceiving synchrony aids in binding of these inputs. In two experiments, we investigated how voluntary movements influence the perception of simultaneity, by measuring simultaneity judgments (SJs) for an audiovisual (AV) stimulus pair triggered by a voluntary button press. In Experiment 1, we manipulated contiguity between the action and its consequences by introducing delays between the button press and the AV stimulus pair. We found a widened window of subjective simultaneity (WSS) when the action-feedback relationship was time contiguous. Introducing a delay narrowed the WSS, suggesting that the wider WSS around the time of an action might facilitate perception of simultaneity. In Experiment 2, we introduced an involuntary condition using an externally controlled button to assess the influence of action-related predictive processes on SJs. We found a widened WSS around the action time, regardless of movement type, supporting the influence of causal relations in the perception of synchrony. Interestingly, the slopes of the psychometric functions in the voluntary condition were significantly steeper than the slopes in the involuntary condition, suggesting a role of action-related predictive mechanisms in making SJs more precise.

## Introduction

Determining whether a clicking sound belongs to a light switch or a keyboard is one of the challenges the brain faces in response to the sensory events around it. Integrating sensory signals is necessary to form a coherent percept, and eventually a meaningful experience of the external world.

Although extensively studied on the perceptual level, research on multisensory interactions mainly focused on sensory stimuli that are external in nature (Alais, Newell, & Mamassian, 2010). However, it has been well-documented that our actions shape how we perceive sensory stimuli compared with when they are spontaneously triggered. One phenomenon regarding this differential processing concerns the perceptual passage of time. In a seminal study by Haggard, Clark, and Kalogeras (2002), it has been shown that there is a subjective compression of time between a voluntary movement and its consequence compared with when a movement is not intentional. More specifically, “the perceived time of the action is shifted forwards towards the effect, and the time of the effect is also shifted backwards in time towards the action that caused it.” (Haggard & Cole, 2007, p. 212).

This effect, known as intentional binding, has been replicated in later studies (Moore & Obhi, 2012; Tsakiris & Haggard, 2003). The tendency to bind voluntary actions to their effects was explained as a process that maintains a sense of agency (Haggard et al., 2002), or perceptual constancy (Yarrow et al., 2001). There is also evidence that intentional binding helps us build causal relations between actions and their consequences (Humphreys & Buehner, 2009). Moreover, a recent study by Kawabe, Roseboom, and Nishida (2013) emphasizes the importance of perceptual grouping between actions and their perception of causally related effects, suggesting that the resulting sense of agency can likely be explained by causality and cross-modal grouping.

Voluntary actions contain a certain sequence of processes to form a coherent stream of conscious action experiences (Haggard et al., 2002). These processes reflect the intention and decision to move, the generation and subsequent execution of the motor command, reafferent sensory feedback, and the use of this feedback for action monitoring (Haggard & Eimer, 1999; Libet, Wright, & Gleason, 1983; K. Yarrow & Obhi, 2014). Interestingly, the perceived timing of actions appears to be different from the actual timing as measured by the cortical activity occurring well before movement onset (the readiness potential; Haggard, Newman, & Magno, 1999; Libet et al., 1983; Obhi, Planetta, & Scantlebury, 2009; Yarrow & Obhi, 2014). If this information is present long before the movement, it could be used to generate predictions that are complete even before the movement occurs. Compatible with this idea, cells have been reported in the parietal cortex that adjust their response properties in anticipation of the postmovement stimulation (Duhamel, Colby, & Goldberg, 1992). The intentional binding effect is not present for involuntary movements or when the consequence of the action follows after an unpredictable delay (Haggard et al., 2002). These findings suggest that our motor system is used to make specific predictions of the effects they produce, influencing timing perception for these effects (Haggard, 2005).

The perception of timing for actions and their effects have mostly been investigated using one sensory modality as the feedback of the action. However, voluntary movements usually have multiple sensory consequences. Tapping a tabletop, for example, creates visual, auditory, tactile, and proprioceptive feedback, which need to be integrated. In general, the nervous system maintains the perception of synchrony between the senses despite variations in the arrival times of sensory inputs to the brain (Fain, 2003). This mechanism is important presumably to determine whether the events are from single or multiple sources (Levitin, MacLean, Mathews, Chu, & Jensen, 2000). Therefore, for example, there is a tendency to perceive two sensory events as simultaneous if they are thought to originate from a common source (Stevenson, Zemtsov, & Wallace, 2012; van Wassenhove, Grant, & Poeppel, 2007; Zampini, Shore, & Spence, 2003).

Very few studies have focused on timing perception in which a voluntary action has multiple sensory consequences. In one study, Parsons, Novich, and Eagleman (2013) assessed recalibration for the perceived timing of audiovisual (AV) stimulus pairs preceded by voluntary button presses. They found that the predictable auditory stimulus was perceived as shifted in time, whereas the perceived timing of the unpredictable visual stimulus remained constant. The authors explain this finding with regard to the prior assumption that sensory consequences of actions should occur without a delay (Stetson, Cui, Montague, & Eagleman, 2006) and that the perceptual system interprets events occurring at short delays after an action as the sensory consequences of that action (Eagleman & Holcombe, 2002). In a recent study, Desantis and Haggard (2016) investigated the influence of action planning and prediction on AV temporal grouping. In the study, participants first learned associations between different simultaneous AV pairs to voluntary button presses or to visual cues. Therefore, both auditory and visual stimuli were predictable by either a movement or a sensory cue. Results showed increased tolerance to asynchronies for AV pairs when they followed previously associated actions. Moreover, they demonstrated that the perception of simultaneity for AV pairs depends on learned timing relations between the action and the outcome of that action.

Another related study by Corveleyn, Lopez-Moliner, and Coello (2015) investigated mechanisms playing a role in sensory binding for action versus perception. Using a perceptual and a motor task, they assessed how judgments of relative timing regarding changes in the color and position of a visual target would differ when they were followed by a voluntary movement. Results indicate reduced temporal asynchrony between color and position changes in the target when they were temporally and spatially close to the movement. The researchers conclude that voluntary actions seem to facilitate binding of sensory events that they trigger, by influencing timing constraints inherent in the neural processing of sensory inputs, leading to a reduction in perceived asynchrony between events.

In both Desantis and Haggard (2016) and Parsons et al. (2013), the focus was on the perceptual grouping of multisensory stimuli when they were triggered by a voluntary movement compared with when they occurred without movement. Similarly, in the Corveleyn et al. (2015) study, the interest was on the perception of changes in intrinsic and extrinsic properties of a target when it was followed by a movement compared with when it was only perceived. In other words, these studies focus more on the influence of voluntary movements on the perception of simultaneity for sensory events, as opposed to when these events occur externally. Therefore, the control conditions in these experiments consisted of passive viewing of the sensory stimuli, which made it possible to assess mere perceptual grouping effects. However, to investigate the effect of action-related predictive processes on the perceptual grouping of sensory inputs, the specific influence of the voluntary movement itself should be considered as well. Voluntary movements lead to both efferent and reafferent feedback. On the other hand, sensory feedback that is externally triggered provides only reafferent information (Weiskrantz, Elliott, & Darlington, 1971).

In the current study, we aimed to investigate how perception of simultaneity was maintained for the multisensory consequences of a voluntary movement. Specifically, we aimed to assess the involvement of temporal contiguity and action-related predictive processing on the perception of simultaneity for multisensory stimuli. For this purpose, we varied the temporal relationship between the action and the multisensory feedback to investigate how changes in temporal contiguity would influence perceived timing of these feedback. In a second experiment, we addressed the influence of action-related predictive processing on the perception of simultaneity for sensory inputs. In both experiments, we asked participants to initiate button presses at a time of their choice. The button press triggered the occurrence of a dot and a tone with a range of stimulus onset asynchronies (SOAs). The participants decided whether the dot and the tone were simultaneous or not. The stimulus pairs were presented either immediately following the button press or with one of two delays. Research on simultaneity perception suggests that there is a tolerance for how far two stimuli can be separated in time and still be perceived as simultaneous. This time window, known as the window of subjective simultaneity (WSS), has been found to be influenced by the assumption that both signals originate from a single source (the “unity assumption”; Vatakis & Spence, 2007). In line with this, we expected to observe an increased tolerance for asynchronies in AV stimuli when they were contiguous with the action. To address the specific influence of voluntary movement, we used an externally controlled button in our second experiment, which allowed us to manipulate the influence of action-related predictive processing on AV simultaneity judgments (SJs). We expected to observe increased tolerance to asynchronies in the voluntary condition compared with the involuntary condition, as the participants would not be in control of the occurrence of AV stimulus pair. Support for this comes from studies showing less or no temporal binding to actions to their outcomes when the actions are not voluntary (Haggard et al., 2002; Tsakiris & Haggard, 2003). This study is the first to assess the influence of action-related predictive processing defined as the absence of intention on simultaneity perception for AV stimuli (representing multisensory consequences of an action).

## Experiment 1

### Methods

#### Participants

Twenty-four right-handed students (mean age 24.1 ± 2.6, 16 females) from Philipps University Marburg took part in the experiment. Informed consent was obtained from all participants included in the study. They reported normal or corrected-to-normal vision and normal hearing. Right-handedness was confirmed by the Edinburgh Handedness Inventory (Oldfield, 1971). The experiment was approved by the local ethics committee and performed in accordance with the Declaration of Helsinki. The participants were paid for their participation.

#### Apparatus

Visual stimuli were presented on a 24” computer screen (1920 × 1200 pixels resolution, 60 Hz frame refresh rate). Auditory stimuli were presented via headphones. Stimulus presentation was controlled by Octave and Psychtoolbox-3 (Brainard, 1997). A chin rest was used to stabilize the subject’s head during the experiment. Button presses were made via a button pad using the participant’s right hand. The button pad was covered with a black box to prevent participants from using visual cues. Responses were made on a keyboard (‘V' for ‘Yes', ‘N' for ‘No') using the left hand.

#### Stimuli and procedure

The visual stimulus was a black dot (1.5° visual angle, 0 cd/m^2^ luminance) at the center of the display against a neutral gray background (∼89 cd/m^2^ luminance). The auditory stimulus was a pure auditory tone burst with a frequency of 250 Hz. To attenuate potential auditory cues arising from the button press, white noise was presented throughout the whole experiment. There were two within-subject factors: SOA and delay. Eleven SOAs between the auditory and visual stimuli were used: ±417.5, ±334, ±250.5, ±167, ±83.5, and 0 ms. Negative SOAs indicate that the auditory stimulus was presented first, whereas positive SOAs indicate that the visual stimulus was presented first. The duration of the first stimulus (dot or tone) was 1000 ms, and stimulus pairs always terminated at the same time. Therefore, the actual duration of the lagging stimulus was smaller than 1000 ms, depending on the SOA. Because we were interested in how movement influences the perception of synchrony, we instructed participants to use event onset times, rather than the offsets. Three delays were presented between the button press and the occurrence of the first stimulus: 0 ms, 417.5 ms, and 2500 ms. A previous study by Haggard et al. (2002) suggested that binding of actions to their effects was stronger when the timing between the action and the consequence was around 250 ms in comparison to 450 or 650 ms, that is, when the stimulus was time contiguous with the action. Another study showed a decreasing tendency to perceive a stimulus as not representing the consequences of a voluntary action after a long delay (Eagleman & Holcombe, 2002; but see Humphreys & Buehner, 2009). We therefore used an intermediate delay of 417.5 ms and a very long delay of 2500 ms. The long delay was expected to serve as a condition in which the action and the effect was torn far apart in time, presumably leading to an impression that the effect did not originate from the action. Each SOA was repeated 10 times for each delay, for 330 trials. The trials were divided into two experimental runs. The combination of SOAs and delays were presented in random order with the restriction that both runs had the same number of delays and SOAs.

The experiment was conducted in a dimly lit room. Participants sat in front of the computer screen at a viewing distance of 54 cm. They were instructed to place their right hand on a button pad, with their index finger on the button. Participants were instructed to perform button presses at a self-chosen time after a cue. The button press triggered the occurrence of the stimulus pair.

The task was to judge whether the dot and the tone were simultaneous or not. Participants were also told that in some trials, there would be a delay between their button press and the occurrence of the stimulus. However, they were told that the task in these trials would remain the same: judging the simultaneity of the dot and the tone. To familiarize participants with the stimuli and the task, participants completed a block of 45 trials with smallest and largest SOAs and delay conditions (5 trials for each combination) with feedback before the start of the experiment. The practice trials were followed by the two experimental runs with a short break in between the runs. The whole procedure took 1.5 hours.

**Figure 1.**
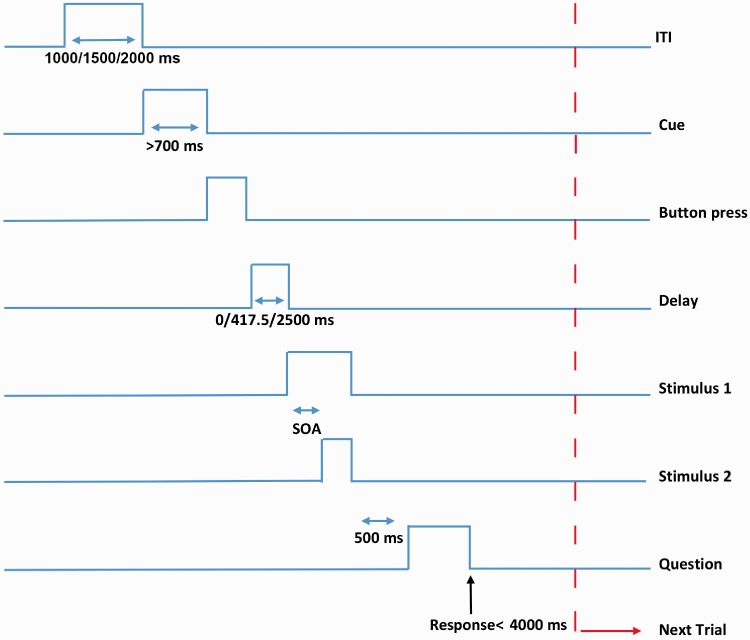
Timeline of an experimental trial in Experiment 1. After a variable ITI, a square cued participants to press a button at a time of their choice. Immediately or after a certain delay, a dot and a tone with variable SOAs (ranging from audition-leading 417.5 ms to vision-leading 417.5 ms) followed. The participants then judged whether the dot and the tone were simultaneous or not. ITI = intertrial interval; SOAs = stimulus onset asynchronies.

Each experimental trial started with a variable intertrial interval (ITI; 1000, 1500, 2000 ms) during which a fixation cross (0.5 × 0.5 cm) was presented. After the ITI, a black square (310 × 310 pixels, 3.2° visual angle) surrounding the fixation cross was presented in the middle of the screen. This square served as a cue for participants to initiate their button press. The participants were instructed to wait for approximately 700 ms after the appearance of the square, but they could choose to wait longer if they wanted. This was done to elicit a well-prepared, self-initiated button press rather than an automatic reflex to the cue (Rohde & Ernst, 2013). If the button was pressed too early, a ‘too early’ warning was presented, and the trial was repeated. After the button press, the multisensory stimulus was displayed following one of the three delays. The square remained on the screen during the presentation of the stimulus pair. After the offset of the stimulus pair and the square, a 500-ms interval followed. Subsequently, the question ‘Simultaneous? Yes/No’ was presented on the screen. Participants used their middle and index fingers of their left hand for responding ‘Yes’ or ‘No’ respectively. They were given a maximum of 4 seconds to respond after which the next trial followed. If they took longer than that, next trial followed. The sequence is shown diagrammatically in Figure 1.

#### Data analysis

We used MATLAB 2012b (The Mathworks Inc., 2012) and psignifit toolbox version 2.5.6 for MATLAB (Fründ, Haenel, & Wichmann, 2011) for plotting SJs and fitting the data. SJs were plotted as a function of SOAs for each participant and delay. The data then were fitted with a two-criterion window model of the SJ task (Cravo, Claessens, & Baldo, 2011; Rohde, Greiner, & Ernst, 2014; Ulrich, 1987; K. Yarrow, Jahn, Durant, & Arnold, 2011; Yarrow, Sverdrup-Stueland, Roseboom, & Arnold, 2013). This model generates a psychometric function that is constructed from the differences of two cumulative probability functions (each having a normal distribution) and has the following four parameters: two means (window limits) representing the positions of the decision criteria on the SOA axis for simultaneity, and two standard deviations (slopes) representing the sums of two sources of variability. The advantage of such a model is that it can account for asymmetries in perceiving simultaneity (Rohde et al., 2014; Yarrow et al., 2011). The values that fall between the decision criteria are considered simultaneous, defining the WSS. In our experiment, the two sides of the psychometric function corresponded to the audition-leading and vision-leading sides, with positive values denoting that the dot came first.

Statistical analyses on the window limits and slopes was carried out using SPSS 21 (IBM Corp. Released 2012, n.d.). We used Huynh–Feldt-corrected degrees of freedom in cases where sphericity was violated as indicated by the Mauchly’s test of sphericity (Huynh & Feldt, 1976).

#### Deviance analysis

To test whether the participants were guessing or found the task difficult, the four-parameter model was tested against a simpler two-parameter model. This simpler model can capture participants who were guessing in which case their data would not vary systematically with SOA, or who were not given a sufficiently broad range of SOAs so that both transitions from synchrony to asynchrony would not be captured. We estimated the deviance for each model fit for each participant in each condition and then calculated the deviance difference (2 × difference in log likelihood) between the models. The difference in deviance from a simpler to a more complex model follows a chi-square distribution with degrees of freedom equal to the number of free parameters between the models (Yarrow, submitted).

We retained participants when this difference was significantly greater than the critical values for the chi-square distribution with 2 degrees of freedom (Yarrow et al., 2013; Yarrow, submitted). None of the participants was excluded on this basis.

### Results

We performed the statistical analyses on the estimated window limits and slopes.

**Figure 2.**
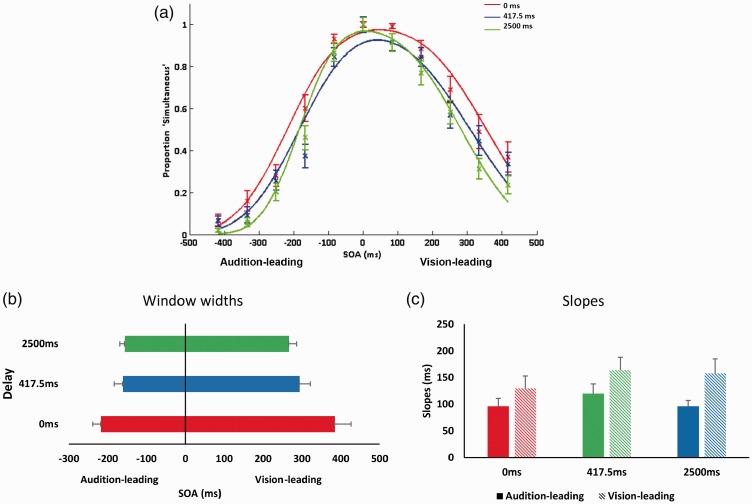
Results from Experiment 1. (a) Proportion of ‘simultaneous’ responses as a function of SOAs for each delay, fitted to a two-criterion window model. Negative SOAs depict trials where the tone came first. Curves are normalized to the peak. The data depictions are provided for illustrative purposes only. Statistical analyses were based on individual data fits. (b) Estimated WSS across all delays, derived from individual fits. (c) Estimated slopes across all delays, derived from individual fits. Error bars denote standard error of the mean. SOAs = stimulus onset asynchronies; WSS = window of subjective simultaneity.

For the statistical analyses on window limits, we first sign-inverted the values on the audition-leading side of the curve to compare their distances from true simultaneity. A 2 (Stimulus order: audition-leading vs. vision-leading) × 3 (Temporal delays: 0 vs. 417.5 vs. 2500 ms) repeated-measures analysis of variance (ANOVA) was conducted on the estimated window limits. There was a main effect of stimulus order, *F*(1, 23) = 39.31, *p* < .001, η^2 ^= 0.35. The window limits for audition-leading side (*M* across all delays = 178, *SD* = 16) were lower than the window limits for vision-leading side (*M* across all delays = 315, *SD* = 25; see Figure 2a). We also found a main effect of delay, *F*(1.32, 30.28) = 13.38, *p* < .001, η^2 ^= 0.11. Bonferroni-corrected post hoc *t* tests showed significant differences between the 0 ms (*M* = 301, *SD* = 27) and 417.5 ms (*M* = 227, *SD* = 22) delays, *t*(23) = 5.87, *p* < .001, *d* = 3.04, and between 0 and 2500 ms (*M* = 211, *SD* = 12) delays, *t*(23) = 3.70, *p* = .001, *d* = 4.36 (see Figure 2b).

A similar repeated-measures ANOVA conducted on the slopes showed a significant effect of stimulus order, *F*(1, 23) = 7.51, *p* < .05, η^2 ^= 0.08. Slopes for the audition-leading side (*M* = 102, *SD* = 13) were significantly steeper than the slopes for the vision-leading side (*M* = 148, *SD* = 19; Figure 2c). The slopes of the curves did not significantly differ across delays.

### Discussion

Participants were highly tolerant of asynchrony between a multisensory stimulus pair when it immediately followed the action. When the temporal interval between the action and the AV pair was increased, there was a greater sensitivity to asynchrony for these stimuli. However, this effect did not increase for the longest delay condition, suggesting that the effect might have saturated. When vision preceded audition, sensitivity to asynchronies was lower (as indicated by higher window limits and shallower psychometric functions) than when audition preceded vision. In other words, decision boundaries to judge the AV pair as simultaneous were higher on the vision-leading side than that of the audition-leading side. Such an asymmetry has been documented previously for AV SJs, in which the PSS was shifted toward a vision-leading stimulus and indicated that the perceived simultaneity is maximal if visual stimulus appears slightly before the auditory stimulus (Dixon & Spitz, 1980; Spence & Squire, 2003; Vatakis & Spence, 2006; Zampini et al., 2003).

Is the widened WSS related specifically to a voluntary movement or just to the occurrence of a stimulus pair immediately following a button press? To test this, we included an involuntary movement condition in which the same task was performed, but with an automatically depressed button. With this condition, we aimed to assess the role of action-related predictions in judging simultaneity while maintaining comparable proprioceptive feedback for both conditions.

## Experiment 2

### Method

#### Participants

Twenty-four participants from Philipps University Marburg took part in the experiment (mean age = 24 ± 3.13, 15 females). They were a different group from the participants in Experiment 1. Informed consent was obtained from all participants included in the study. They reported normal or corrected-to-normal vision and normal hearing. Right-handedness was confirmed by the Edinburgh Handedness Inventory (Oldfield, 1971). The experiment was approved by the local ethics committee and performed in accordance with the Declaration of Helsinki. The participants were paid for taking part in the experiment.

#### Apparatus

The setup was the same as in the first experiment, except that a custom-made button was used. This allowed us to actively pull down the button with an electromagnet (Intertec, ITS-LS2924B-D, 12 V DC). Stroke length of the button was 5 mm with a micro switch triggered within the last 0.2 mm of movement. Both voluntary manual and externally activated button presses were recorded by the computer as a left click of a USB mouse. Therefore, jitter and delay of the button press did not depend on whether the condition was voluntary or involuntary. For the voluntary movement, the initial force was 1.5 Newton (N), as measured by a spring force gauge, slowly increasing to approximately 2.5 N in the final position. In the involuntary movement condition, the finger was initially pulled with approximately 1 N, and the force increased to approximately 4 N in the final position. The duration of the depression of the button press in the involuntary condition was set to 300 ms that was representative of the duration of the button press based on a previous piloting.

The AV stimulus pair was presented only when the button was depressed completely, both for voluntary and involuntary conditions. Participants wore soft foam earplugs to attenuate the sound of the ‘involuntary’ button press. Moreover, white noise was presented to attenuate any sound, especially the one caused by the externally activated button. The participant’s right index finger was fixed to the button by a cotton bandage that was flexible enough not to cause discomfort. This was done to ensure that the finger would go along with the button in the involuntary condition. The cotton bandage was present in both voluntary and involuntary conditions.

#### Stimuli and procedure

The stimuli were identical to the previous experiment with the following differences: The 2500 ms delay condition in the first experiment was not found to be different than the intermediate delay condition. For that reason, we excluded the longest delay condition and included an intermediate delay between 0 and 417.5 ms delays. The following delays were used: 0, 167.7, and 417.5 ms.

The procedure was identical to Experiment 1, except for the following: In the voluntary condition, participants made button presses at a self-paced time, with the same timing (>=700 ms) criterion as in Experiment 1 following the start of the cue. In the involuntary condition, the button was pulled down automatically by the computer after the appearance of the cue. The timing of the automatic button press was jittered across trials (700–1500 ms). This was done to prevent participants from predicting the timing of the button press from the cue. The participants were presented with both voluntary and involuntary conditions divided into 2 runs on 2 consecutive days. The order of voluntary and involuntary conditions were counterbalanced across participants. The entire procedure took 1.5 hours.

#### Data analysis

The analysis was the same as in the previous experiment. One participant completed only the first half of the study, so we excluded the data from this participant. We analyzed the data from the remaining 23 participants (mean age = 23.57 ± 3.34, 14 females). The data were plotted and fitted to the two-criterion model. Deviance analyses showed that three participants had one condition that did not conform to the chi-square distribution. We excluded all the data from these participants from the final analyses.

### Results

**Figure 3.**
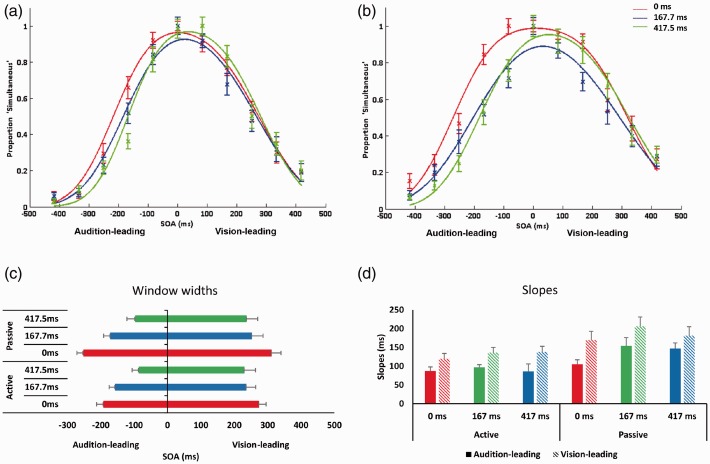
Results from Experiment 2. (a) and (b) Proportion of ‘simultaneous’ responses as a function of SOAs for each delay, fitted to a two-criterion window model, for voluntary and involuntary conditions, respectively. Negative SOAs depict trials where the tone came first. Curves are normalized to the peak. The data depictions are provided for illustrative purposes only. Statistical analyses were based on individual data fits. (c) Estimated WSS across all delays, collapsed across voluntary and involuntary conditions, derived from individual fits. (d) Estimated slopes across all delays, collapsed across voluntary and involuntary conditions, derived from individual fits. Error bars denote standard error of the mean. SOAs = stimulus onset asynchronies; WSS = window of subjective simultaneity.

A 2 (Movement type: voluntary vs. involuntary) × 2 (Stimulus order: audition-leading vs. vision-leading) × 3 (Temporal delays: 0 vs. 167.7 vs. 417.5 ms) repeated-measures ANOVA was conducted on the estimated window limits. There was no main effect of movement type, *F*(1, 19) = 3.50, *p* = .08. We found a main effect of stimulus order, *F*(1, 19) = 17.35, *p* < .01, η^2 ^= 0.23. Thus, we replicated our previous results on asymmetrical window limits for simultaneity when vision led audition (*M* = 257, *SD* = 26) in comparison to when audition led vision (*M* = 159, *SD* = 15; Figure 3a and b). There was also a main effect of delay, *F*(1.88, 35.68) = 34.75, *p* < .001, η^2 ^= 0.14. Bonferroni-corrected post hoc *t* tests showed that there was significant differences across all the delay conditions: between 0 (*M* = 257, *SD* = 16) and 167.7 ms (*M* = 205, *SD* = 21), *t*(19) = 5.88, *p* < .001, *d* = 3; between 167.7 and 417.5 ms (*M* = 163, *SD* = 21), *t*(19) = 3.56, *p* = .002, *d* = 2.05; and between 0 and 417.5 ms, *t*(19) = 7.25, *p* < .001, *d* = 5.31 (Figure 3c). We also found a significant interaction between stimulus order and delay, *F*(1.62, 30.71) = 8.07, *p* < .01, η^2 ^= 0.02. Bonferroni-corrected post hoc *t* tests showed that in the audition-leading side of the curve, the window limits were significantly higher for the 0 ms (*M* = 192, *SD* = 90) compared with the 167.7 ms (*M* = 157, *SD* = 78) delay, *t*(19) = 4.89, *p* < .001, *d* = .08; the 167.7 ms compared with the 417.5 ms (*M* = 86, *SD* = 89) delay, *t*(19) = 4.54, *p* < .001, *d* = 0.09; and the 0 ms compared with the 417.5 ms delay, *t*(19) = 9.73, *p* < .001, *d* = 1.67. For the vision-leading side, the window limits were significantly higher for the 0 ms (*M* = 293, *SD* = 102) compared with the 167.7 ms (*M* = 244, *SD* = 131), *t*(19) = 3.75, *p* = .001, *d* = 0.42; and the 0 ms compared with the 417.5 ms (*M* = 234, *SD* = 134), *t*(19) = 3.53, *p* = .002, *d* = 0.51.

A similar repeated-measures ANOVA was conducted on the slopes. There was a main effect of movement, *F*(1, 19) = 30.78, *p* < .001, η^2 ^= 0.09 (Figure 3d). The slopes for the voluntary condition (*M* = 118, *SD* = 7) were significantly steeper the slopes for the involuntary condition (*M* = 160, *SD* = 11). We also found a main effect of delay, *F*(1.46, 27.76) = 4.51, *p* < .05, η^2 ^= 0.03. Bonferroni-corrected post hoc *t* tests showed that the slopes were significantly steeper for 0 ms (*M* = 120, *SD* = 11) compared with the 167.7 ms delay (*M* = 148, *SD* = 11), *t*(19) = 4.25, *p* < .001, *d* = 2.58, and the 0 ms in comparison to the 417.5 ms delay (*M* = 148, *SD* = 10), *t*(19) = 2.11, *p* = .049, *d* = 2.72. We also found a main effect of stimulus order, *F*(1, 19) = 7.28, *p* < .01, η^2 ^= 0.07. The slopes for the audition-leading side of the curve (*M* = 119, *SD* = 9) were significantly steeper than the slopes for the vision-leading side of the curve (*M* = 158, *SD* = 13), which was again a replication of the asymmetry.

### Discussion

In the second experiment, we found increased tolerance for AV asynchronies around the time of action, replicating our previous finding. This was also the case for the involuntary condition, suggesting that the contribution of action-related predictive mechanisms, which we expected to be much better for the voluntary condition, did not in fact have an effect on the tolerance for SOAs in simultaneity perception. Delaying the presentation of the stimulus pairs after the movement led to decreased tolerance for asynchronies both for voluntary and involuntary movements.

The slopes of the psychometric functions were significantly steeper for the voluntary in comparison to the involuntary condition. This finding suggests that the participants’ responses in the involuntary condition were more variable. Moreover, the difference in slopes with the inclusion of delays suggests that overall SJs had more variance in the presence of delays. We replicated our finding of asymmetrical window limits and slopes for the audition- and vision-leading sides of the SJ curve.

## General Discussion

In the present experiments, we investigated the perception of simultaneity for multisensory stimuli triggered by voluntary or involuntary movements and compared SJs when the timing between action and the feedback was and was not contiguous. The WSS widened when stimuli were contiguous with the action. Introducing a delay between the action and the AV feedback increased sensitivity for AV asynchrony. In other words, the decision boundaries for judging the stimuli as simultaneous were significantly wider when AV stimuli immediately followed the button press than when a delay was added. Introducing a delay of 2500 ms, which eliminated contiguity for the action-effect relationship, produced data that were not different from introducing 417.5 ms delay, suggesting that 417.5 ms was enough to break contiguity. The similarity between the voluntary and involuntary conditions suggests that perceiving simultaneity between the AV stimuli was not affected by action-related predictive mechanisms. However, the psychometric slopes were steeper when participants pressed the button voluntarily as compared to when the movement was involuntarily executed. We now discuss these findings in detail.

There are a number of possible explanations with regard to our finding of increased tolerance of SOA in the perception of simultaneity when the action was time contiguous with AV feedback: First, the participants could have become more likely to perceive both components as due to their action, and therefore should be linked, which then might have led to a shift in the perceived timing of sensory stimuli toward the action. Second, time could be perceived as slowed around the point where the action was executed, and therefore it was harder to make any kind of temporal judgment. The two explanations presumably lead to intentional binding of an action to its consequences. However, each explanation underlies a different type of processing of time around the occurrence of an action (Haggard et al., 2002; Morrone, Ross, & Burr, 2005). The first explanation is related to timing shifts toward an action. According to this hypothesis, the subjective timing of anticipated action-effect intervals is shifted backward in time toward the action, which leads to perceived shortening of action-effect intervals (Wenke & Haggard, 2009). In our experiment, we found that the WSS were increased for the AV stimulus pair when it immediately followed the movement, suggesting a possible time shift for the second sensory stimulus relative to the first sensory stimulus that was presented immediately following the action. An alternative explanation could be the compression of perceptual time around the point of an action (Morrone et al., 2005; Wenke & Haggard, 2009). Accordingly, operant movements such as voluntary actions cause compression of time around the movement, which is thought to stem from a slowed internal clock speed during the movement. This leads to compressed intervals between the action and the effect. In this respect, the widened WSS around the time of the action in our experiment might correspond to perceptual time slowing down and events seeming to be closer together in time than they normally are. Although our finding of poor temporal discrimination around a voluntary movement cannot be directly attributed to time shift or compression, it suggests that participants are highly tolerant of asynchrony between a multisensory stimulus pair when they immediately followed the action. Such tolerance might facilitate perception of synchrony between multisensory action outcomes. The findings of our study clearly highlight the differential contribution of voluntary movements in perceiving multisensory inputs and is in line with the literature on the differential processing of sensory information in relation to voluntary movements (Corveleyn et al., 2015; Desantis & Haggard, 2016; Parsons et al., 2013). As in these studies, our findings similarly indicate a reduction of temporal asynchrony between sensory events attributed to an action. In addition, including delays between a movement and its consequences resulted in a similar increase in perceiving asynchronies. These results in general underlie the complex relationship between action and perception, adapted to the demands of the world we are interacting with. Supporting this view comes from research on peripersonal space (PPS). PPS, defined as the region around the body, is important for survival as harmful stimulus poses a more likely threat when it is near the body, and our interactions with the external world usually occurs within this space (Brozzoli, Gentile, Petkova, & Ehrsson, 2011; Graziano & Cooke, 2006). When considered in the context of our study, PPS provides an important clue to the mechanisms underlying the prioritized processing of sensory inputs near us or those inputs resulting from our own action. A recent study by Noel, Lukowska, Wallace, and Serino (2016) addressed this issue by investigating how perception of simultaneity for an AV event is influenced by whether they were within or outside the person’s PPS. By manipulating the distance of AV events to the body (inside or outside PPS), they found significantly higher SJs when the stimuli were within the PPS. The study provides additional support to the finding that a more liberal binding criteria within the context of PPS is advantageous for an efficient processing of sensory information being in close proximity to the body, where interaction with the external world is more likely (Brozzoli, Gentile, & Ehrsson, 2012; Graziano & Cooke, 2006; Rizzolatti, Fadiga, Fogassi, & Gallese, 1997). Our findings regarding wider WSSs around a movement are in line with Noel et al. (2016), as both studies support flexible criteria for binding sensory information when interacting with and reacting to the world.

Despite our finding regarding timing distortions linked to voluntary movements, larger binding windows for AV stimulus pairs were found around the time of involuntary movements as well. An alternative explanation of our findings therefore involves the assumed causal relations between events. Following from a Bayesian view, there is a general prior assumption that causally related events are more likely to occur close in time and space (Eagleman & Holcombe, 2002; Hume, 1748). For example, Buehner (2012) demonstrated that predictive relations were constructed not only for voluntary movements or intentional agents but also when a machine caused the event. However, in contrast, there is also evidence that involuntary movements lead to less binding than voluntary movements (Tsakiris & Haggard, 2003; Wohlschläger, Haggard, Gesierich, & Prinz, 2003). There are studies suggesting that both intentionality of the movement as well as causal relations between the events are important (Cravo, Claessens, & Baldo, 2009; Cravo et al., 2011; J. Moore & Haggard, 2008). Our data are more in line with this view as increased WSS for AV pairs were both present for voluntary and involuntary conditions, suggesting that contiguity between the button press and the AV stimuli led to an increased distortion of time for these events, both when the participants were voluntarily initiating them as well as when they were mechanically initiated. This view still predicts attraction between causally related events outside of one’s own control (Eagleman & Holcombe, 2002). In both voluntary and involuntary cases, a causal relationship between the button press and the appearance of an AV pair was present. Nevertheless, an interesting finding emerged in the form of steeper slopes for the voluntary condition, suggesting that the participants were more precise in their judgments when they were initiating the button press. This points to the differential processing for the consequences of voluntary movements and is in line with the finding that people are more confident about events that they caused (Stetson et al., 2006). Therefore, when the participants voluntarily initiated the movement, they were more confident that the AV feedback was simultaneous than when the button press was involuntarily initiated.

A possible implication for the increased WSS in our study is related to the assumption of unity in perceiving synchrony. It has been claimed that intentional binding might contribute to the assumption of unity, which is a prerequisite for integrating sensory signals (Rohde & Ernst, 2013; Rohde et al., 2014). For two events to be bound, they should fall within a window of integration (Bresciani et al., 2005; Shams, Kamitani, & Shimojo, 2000). Following from this, we could argue for our study that this prior assumption was present, and that the perception of simultaneity was maintained over a range of asynchronies for stimuli that were causally linked to a button press be it a voluntary or an involuntary event.

It should be pointed out that in our study, self-generated stimuli were predictable in time, while the comparison stimuli occurred at unpredictable times. This could be viewed as a plausible and sufficient comparison condition considering the real-world situations where involuntary movements are often unpredictable. However, in their review, Hughes, Desantis, and Waszak (2012) point to mechanisms other than motor prediction that might influence the sensory processing of action effects. Among them is temporal prediction, which is defined as “the ability to predict the point in time at which a sensory event will occur” (p. 135). In this sense, a stimulus can be predictable in time when it follows a voluntary or an involuntary movement, so long as it occurs at a specified point in time after the movement. In our experiment, window limits were similar for the voluntary and involuntary conditions, but the judgments were noisier in the involuntary condition (shallower slopes). This increase in noise could reflect the fact that stimuli were less precisely predictable in the involuntary condition than they were following a voluntary button press.

Research on multisensory binding consistently shows that the brain adapts to the prolonged exposure of asynchronous multisensory stimuli to compensate for environmental influences as well as differences in the speed of neural processing (Fujisaki, Shimojo, Kashino, & Nishida, 2004; Vroomen, Keetels, De Gelder, & Bertelson, 2004). Recently, it has been demonstrated that adaptation can take place in a rapid fashion, even without consciously attending to the temporal relations between sensory inputs (Harvey, Van der Burg, & Alais, 2014; Van der Burg, Alais, & Cass, 2013; Van der Burg & Goodbourn, 2015; Van der Burg, Orchard-Mills, & Alais, 2015). Therefore, we assessed whether our results could be explained by rapid recalibration in post hoc analyses. We found no indication of such an effect and therefore ruled out the possible impact of recalibration in explaining our results. However, the effect of rapid recalibration would be an interesting topic for future studies.

It should be noted that although duration of a stimulus is not indicated as a significant factor affecting synchrony perception (Vatakis & Spence, 2006), differences in the duration of auditory and visual stimuli have been found to influence judgments of synchrony (Kuling, van Eijk, Juola, & Kohlrausch, 2012). This effect is not seen when the durations of both stimuli were matched. In our study, the durations of the visual and auditory stimuli changed, so as to encourage participants to attend to the onsets, and not offsets in judging simultaneity. As a result, depending on the SOA, different durations of auditory and visual stimuli were presented. Although it has been shown that increases in the absolute duration of multisensory stimuli lead to decreases in intersubject variability of PSS (Boenke, Deliano, & Ohl, 2009; Kuling et al., 2012), future studies are needed to address the complementary effects of duration on the perception of synchrony.

Another point concerning the length of stimuli is the possibility that participants in our study made use of durations rather than onsets in judging simultaneity. There is evidence that intervals with an auditory onset are perceived to be longer than intervals with an auditory offset (Grondin & Rousseau, 1991; Ortega, Guzman-Martinez, Grabowecky, & Suzuki, 2014; Zampini, Brown, et al., 2005; Zampini, Guest, Shore, & Spence, 2005). However, this effect is found to occur if the onsets and offsets of the auditory and visual stimuli are in close temporal proximity to each other (Chen & Yeh, 2009; Klink, Montijn, & van Wezel, 2011; Romei, De Haas, Mok, & Driver, 2011). In the present studies, although stimulus offsets were always the same, the onsets were mostly incongruent. In this sense, the impact of auditory stimulus on judging simultaneity might not be an explanation for our finding as incongruencies were present more than congruencies. In addition, a recent study by Linares and Holcombe (2014) regarding the perception of latency for AV stimuli indicates that the asymmetric criterion present in judging simultaneity is not present for duration judgments. Considering this and other results on the auditory capture of duration, we believe that if the decisions were based more on duration rather than on simultaneity, window widths and slopes for audition- and vision-leading sides would be similar. Instead, we observed significant differences regarding window widths and slopes of the audition- and vision-leading sides, suggesting simultaneity to be more relevant for the participants in performing the task.

In conclusion, our study shows increased tolerance for asynchronies between AV stimuli around the time of an action that might facilitate binding of sensory signals and compensate for incongruent timing between the senses. A similar pattern emerged for involuntary movements, underlying the importance of causal relations between events. Nevertheless, a unique contribution of action-related predictions in perceiving simultaneity for events emerged with increased precision for judging simultaneity when they follow voluntary movements.
